# Supplementary material for: 1,3-Dioxolan-4-Ones as Promising Monomers for Aliphatic Polyesters: Metal-Free, in Bulk Preparation of PLA
Source: Polymers (Basel). 2020 Oct 18;12(10):2396. doi: 10.3390/polym12102396 (PMC7603121; doi:10.3390/polym12102396)

1,3-Dioxolan-4-Ones as Promising Monomers for Aliphatic Polyesters: Metal-Free, in Bulk Preparation of PLA

Stefano Gazzotti [1,2,*] Marco Aldo Ortenzi [1,2], Hermes Farina [1,2] and Alessandra Silvani [1,2]

[1] Department of Chemistry, University of Milan, Via Golgi 19, 20133 Milan, Italy; marco.ortenzi@unimi.it (M.A.O.); hermes.farina@unimi.it (H.F.); alessandra.silvani@unimi.it (A.S.)

[2] CRC Materiali Polimerici (LaMPo), Department of Chemistry, University of Milan, Via Golgi 19, 20133 Milan, Italy

* Correspondence: stefano.gazzotti@unimi.it; Tel.: +39-02-50314135

Received: 24 September 2020; Accepted: 15 October 2020; Published: date

Supporting Information

INDEX

1. ^{1}H NMR spectra S2-S3
2. DSC Analyses S4
3. TGA Analysis S5
4. GPC Analyses S6

^{1}H NMR (400 MHz, $CDCl_3$) of **1,3-dioxolan-4-one (MeDOX)**

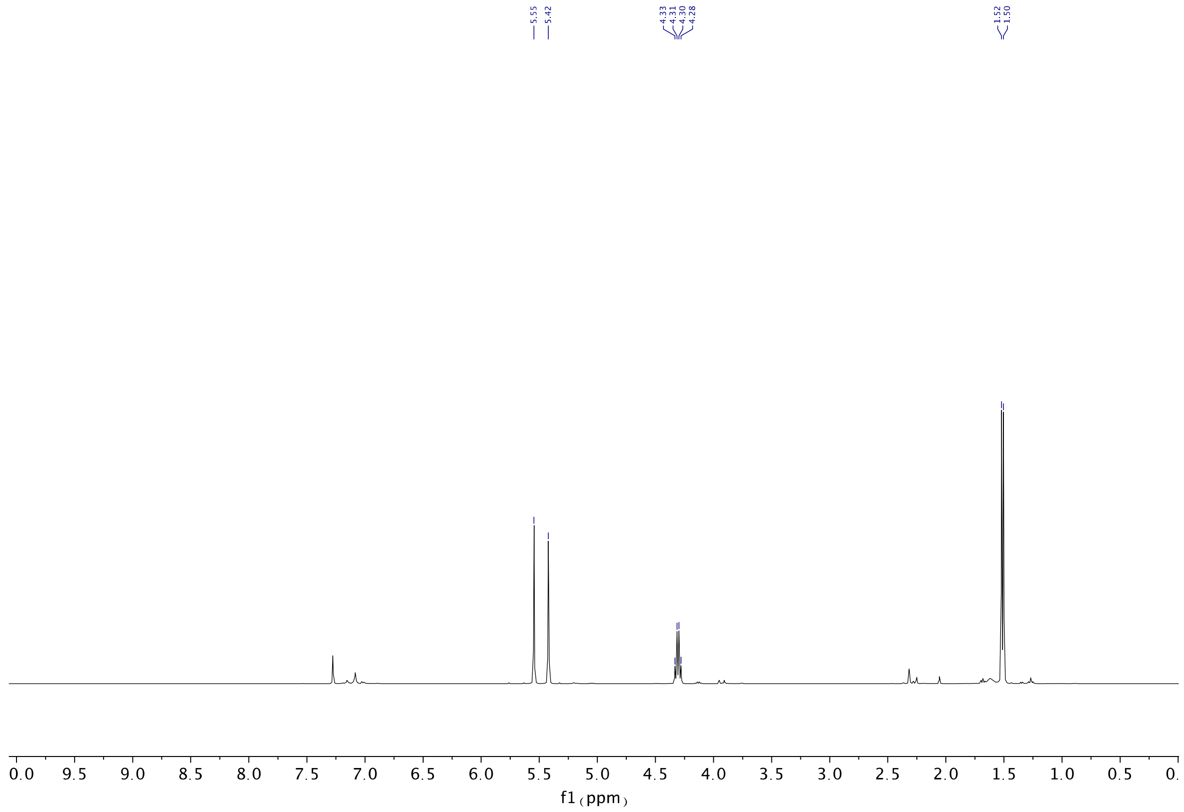

^{1}H NMR (400 MHz, $CDCl_3$) of **2,2,5-trimethyl-1,3-dioxolan-4-one (Me_3DOX)**

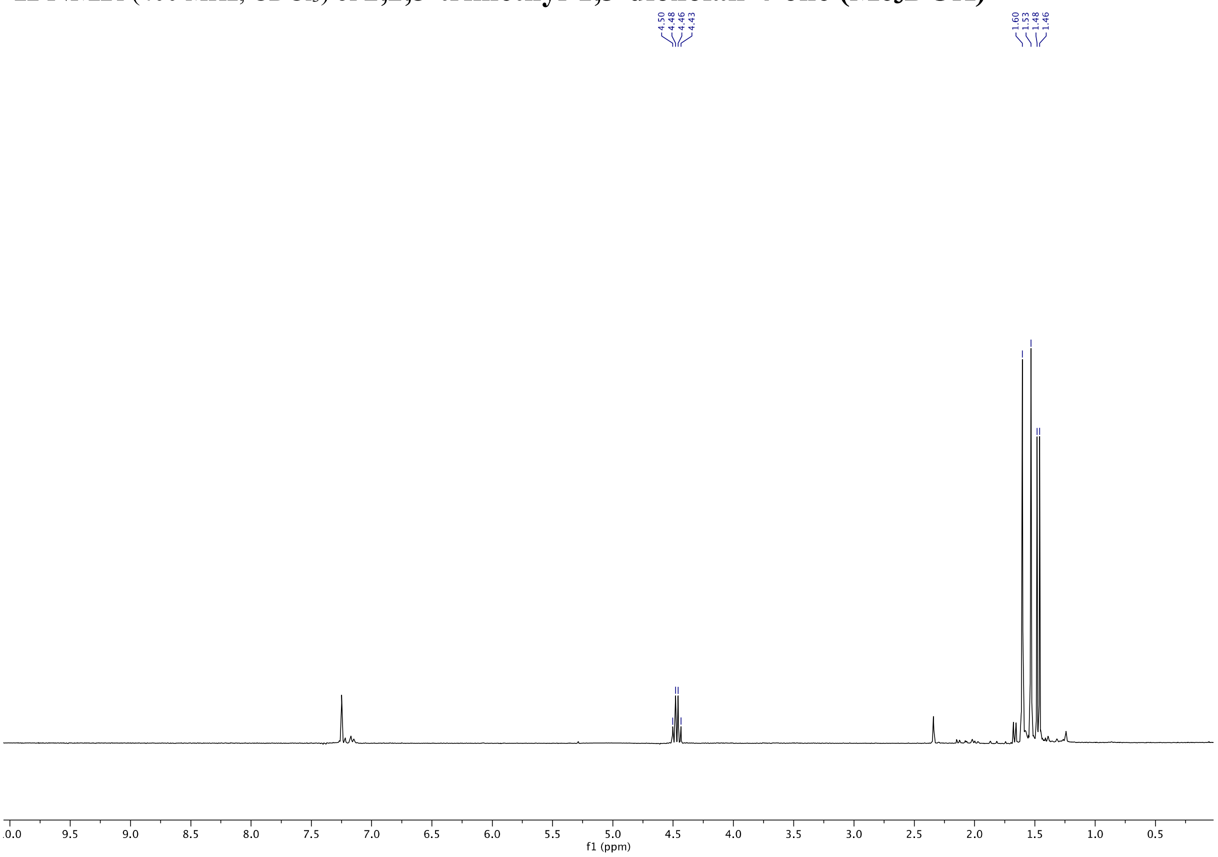

^{1}H NMR (400 MHz, $CDCl_3$) purified polymerization product.

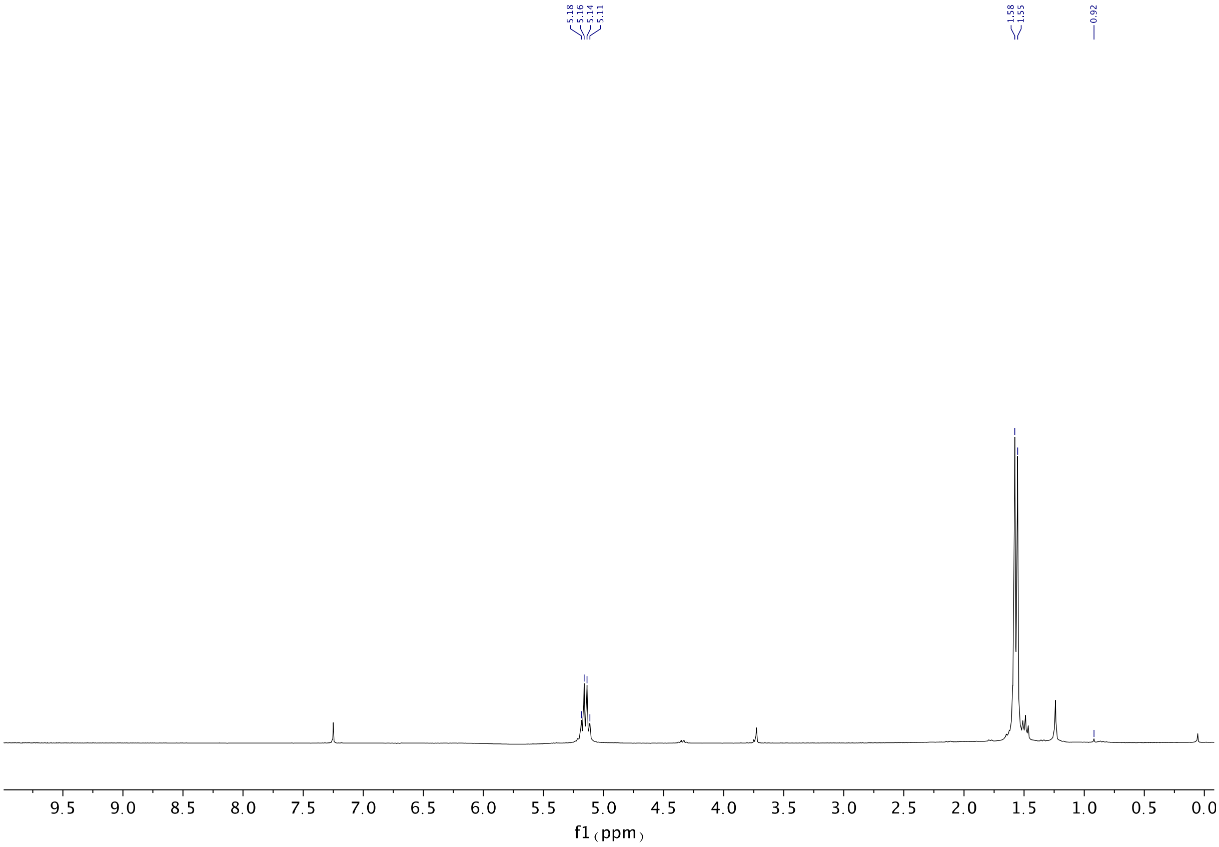

^{1}H NMR (400 MHz, $CDCl_3$): expansion. t-Butyl signal relative to neopentanol initiator highlighted.

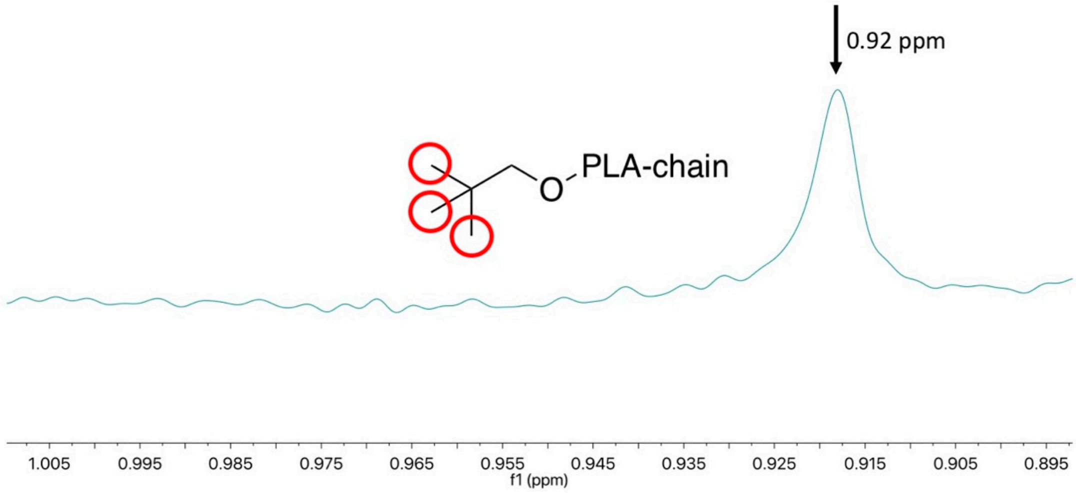

DSC analysis

DSC thermogram

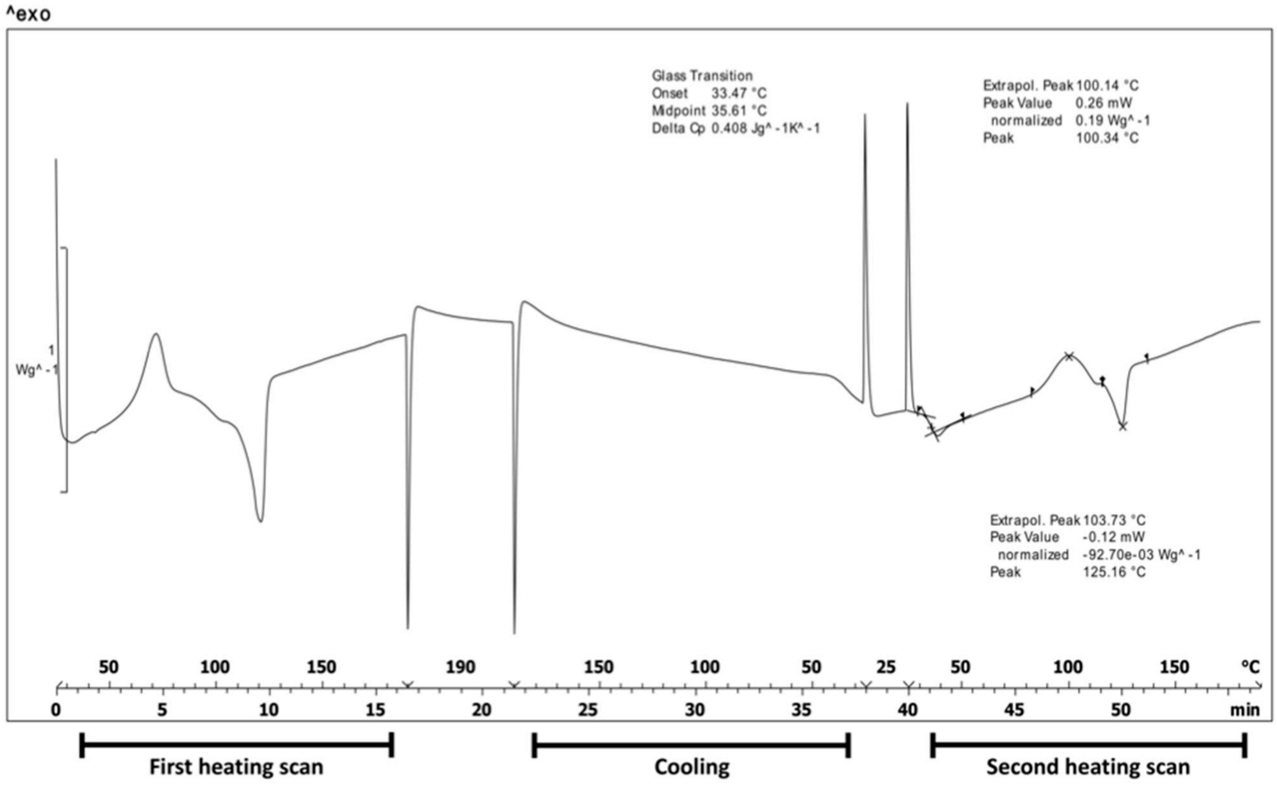

Magnification of the second heating scan in the DSC thermogram is reported below.

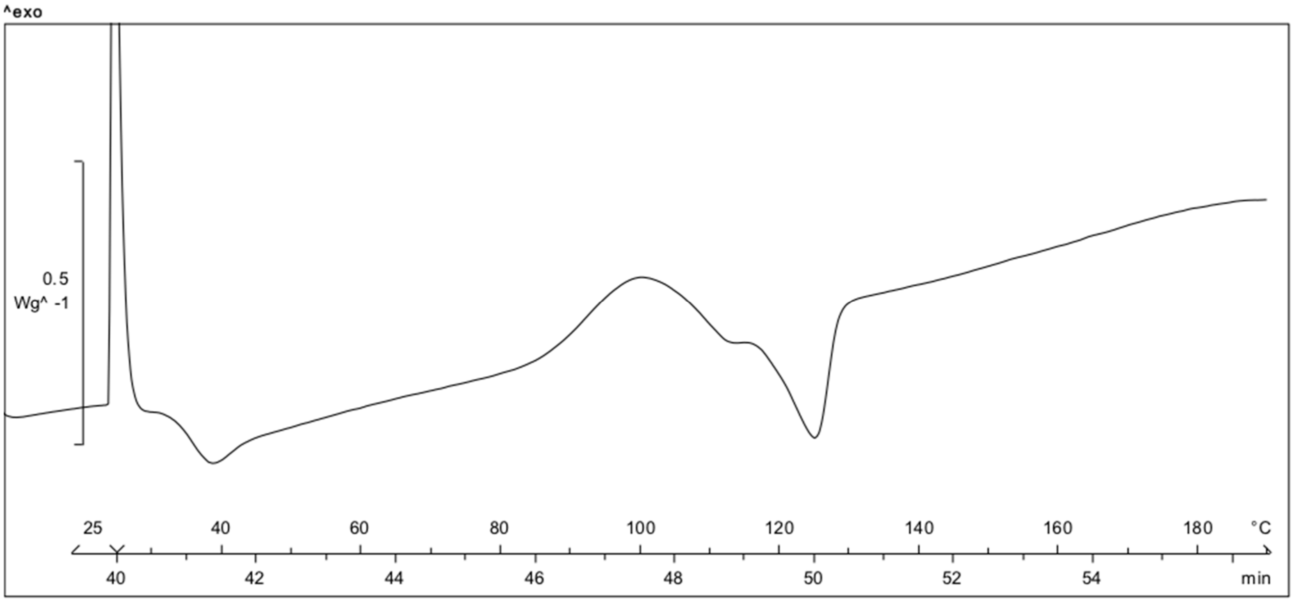

TGA analysis

TGA curve relative to the purified product is reported below.

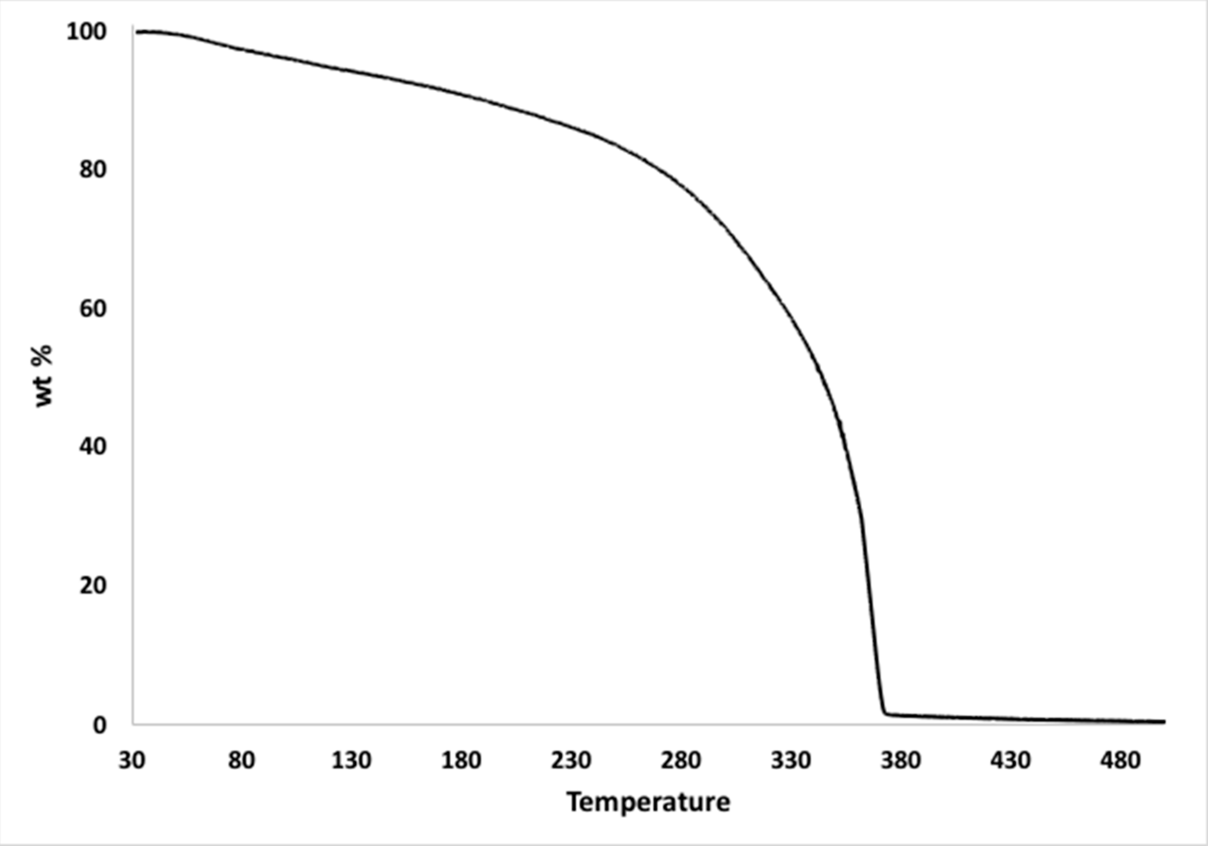

SEC analyses

SEC chromatograms relative to samples taken at different times from Me_3DOX polymerization reaction. Curves have been normalized with respect to the polymer peak, when possible. Given the presence of a high quantity of oligomers and acetone self-condensation by-products, the UV signal doesn't get back to low values after the elution of the main peak.

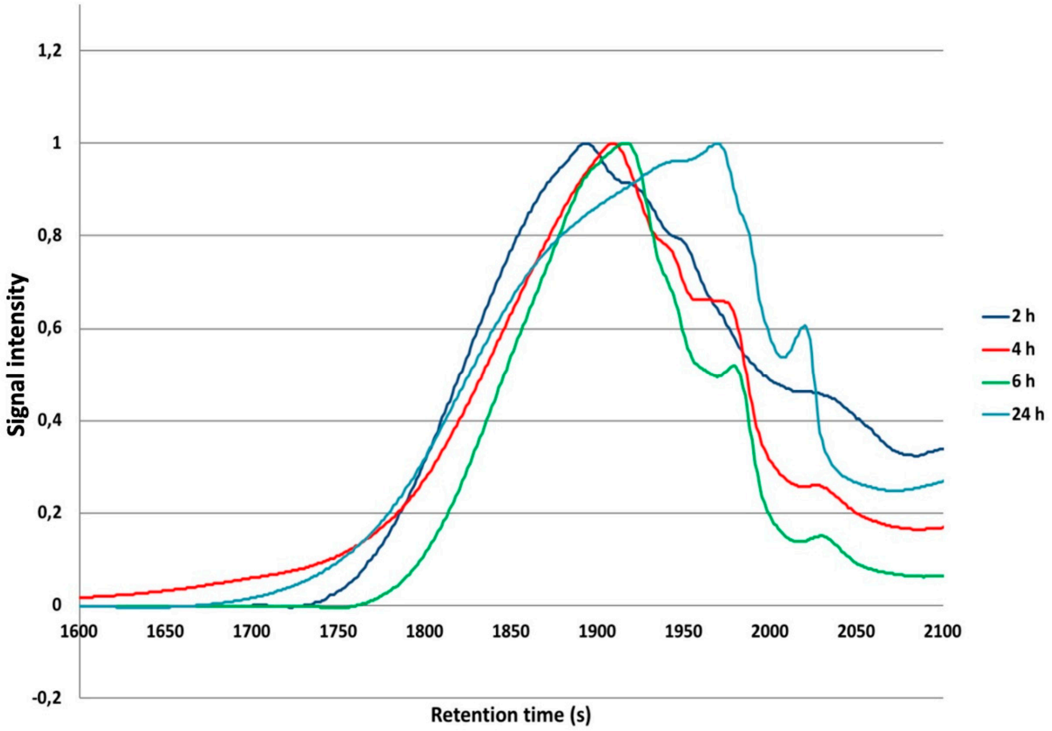

Supplement: Supplementary file 1 [file polymers-12-02396-s001.pdf]
